# Recent changes in daily climate extremes in an arid mountain region, a case study in northwestern China’s Qilian Mountains

**DOI:** 10.1038/s41598-017-02345-4

**Published:** 2017-05-22

**Authors:** Pengfei Lin, Zhibin He, Jun Du, Longfei Chen, Xi Zhu, Jing Li

**Affiliations:** 1 Linze Inland River Basin Research Station, Chinese Ecosystem Research Network, Key Laboratory of Eco-hydrology of Inland River Basin, Northwest Institute of Eco-Environment and Resources, Chinese Academy of Sciences, Lanzhou, 730000 China; 2 University of Chinese Academy of Sciences, Beijing, 100049 China

## Abstract

Changes in climate extremes pose far-reaching consequences to ecological processes and hydrologic cycles in alpine ecosystems of the arid mountain regions. Therefore, regional assessments in various climates and mountain regions are needed for understanding the uncertainties of the change trends for extreme climate events. The objective of this study was to assess the spatial distribution and temporal trends of extreme precipitation and temperature events responses to global warming on the arid mountain regions of China. Results found that temperature extremes exhibited a significant warming trend, consistent with global warming. Warming trend in autumn and winter were greater than in spring and summer. Besides, precipitation extremes also exhibited statistically increase trend, such as number of days with heavy precipitation and rain day precipitation, etc. The distribution of the number of rainy days was showed a significant increasing trend in many sites, indicating that the increase of rain day precipitation mainly contributed by the increase of single precipitation event duration and moderate-rain days. The greater increasing trend of extreme climate events mainly existed in higher altitudes. This results lend an evidence to earlier predictions that the climate in northwestern China is changing from cold-dry to warm-wet.

## Introduction

Climate extremes play an important role in influencing society and natural systems. Given their importance and the uncertainty for prospect of changes in the future, it is very important to understand how and why climate extremes have changed in the past^1^. Thus, extreme climatic events have become of special interest worldwide due to global warming. Changes in, and consequences of temperature and precipitation extremes have been observed at higher rates during the last few decades than earlier, such as heatwave, high temperature, and multi-region heavy rainfall and flood^2–10^. These changes resulted not only in direct increases in the annual mean temperatures and precipitation, but also in the frequency and intensity of extreme climatic events, such as high temperatures, rainstorms, and floods. Consequences for society have risen, and included direct or indirect life and property losses due to extreme climatic events^11^. Consequences of extreme climate events for natural systems included extension of the growing season^3^, increased incidence of spring drought events^12^, and adverse effects on soil erosion processes^13^. Thus, a detailed analysis of the spatial and temporal characteristics of temperature and precipitation extremes is essential for establishing ecosystem management strategies for a changing climate. For example, the number of frost days, duration of warm spells, and changes to the growing season length are important to vegetation growth, water management, and public health^14^.

The Qilian Mountains in northwestern China are typical of the country’s arid mountain regions. Ecological processes and hydrologic cycles in the Qilian Mountains are closely coupled to atmospheric influences and may be highly sensitive to climate change^15^. High relief and high gradients make mountain ecosystems vulnerable to slight changes in temperature and to extreme precipitation events^16^, because variations in temperature constitute the most stressful growth-limiting factors, and a challenge for stability and resilience of plant growth^17^. Spatial or temporal shifts in precipitation regimes would heavily impact the river systems originating in mountain areas, leading to disruptions of the socio-economic structures of people living in the mountains and downstream^18^. The Qilian Mountains are the source of several semi-arid inland rivers, including the Heihe, Shiyang, and Shule. The annual water yield volume entering the Heihe River is critical to people’s livelihood downstream^15^, because the Heihe flows through a region of irrigated agriculture and into two lakes in the desert.

Recent research revealed that growing-season temperatures were an important limiting factor for tree-ring growth in the Qilian Mountains^19^, while temperature and precipitation controlled the distribution and stocks of soil organic carbon^20, 21^. In the past 50 years, the average temperature has risen by 0.26 °C / decade in this region, higher than the national rate of 0.14 °C / decade^22^. Moreover, the frequency of extreme climatic events (e.g. high temperatures and rainstorms) increased in recent years^23^. Previous studies on climate change in the Qilian Mountains have concentrated on average temperature and average precipitation series^22, 24, 25^. A detailed analysis of the spatial and temporal variability in temperature and precipitation extremes will further our understanding of climate-change effects on ecological processes and hydrologic cycles in this region.

In this paper, we focused on spatial and temporal variability in daily climate extremes in an arid mountain region to (1) provide a regional assessment of extreme climatic events from 1960 to 2014 for the Qilian Mountains by analyzing the annual spatial and temporal trends in temperature and precipitation extremes, and (2) examine linkages between the extremes and average annual temperature and precipitation.

## Results

**Figure 1:**
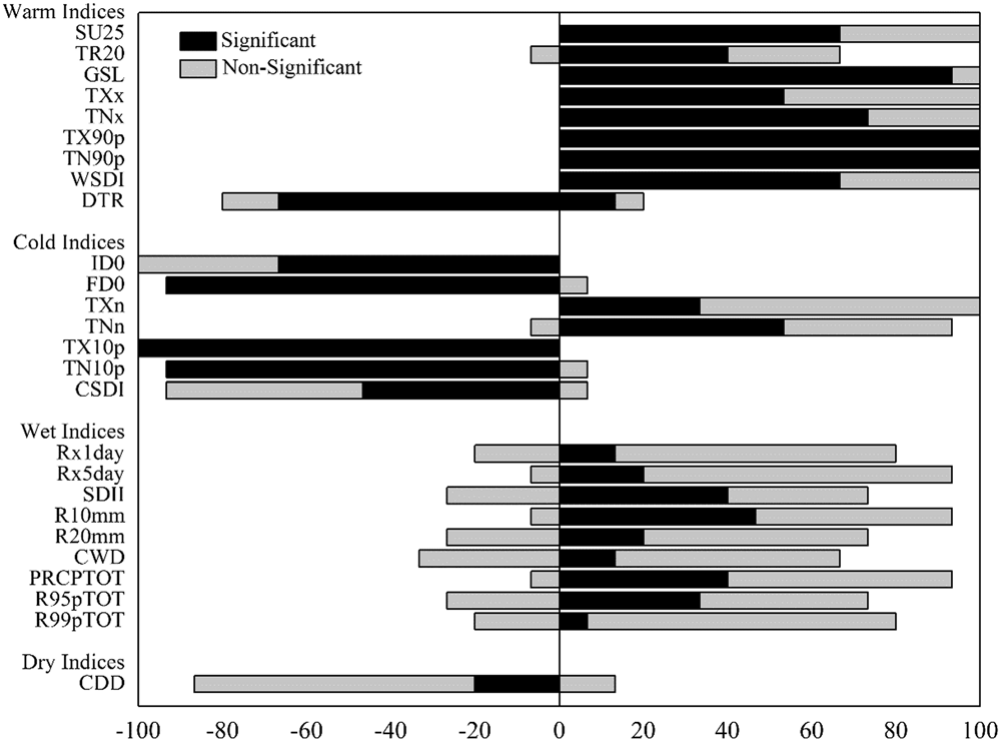
Percentage of stations showing positive and negative trends for indices of climate extremes. For description of index names, see Supplementary Table S1. Black represents significant at the 5% level and gray represents no significant. 0–100 shows an increasing trend, −100–0 shows a decreasing trend.

The summary of positive and negative trends for the calculated indicators of climate extremes is shown in Fig. 1. Each indicator was assessed based on consistency of the trend direction and the proportion of stations exhibiting such trends, significant at the 5% level.

### Spatial trends in temperature extremes

**Figure 2:**
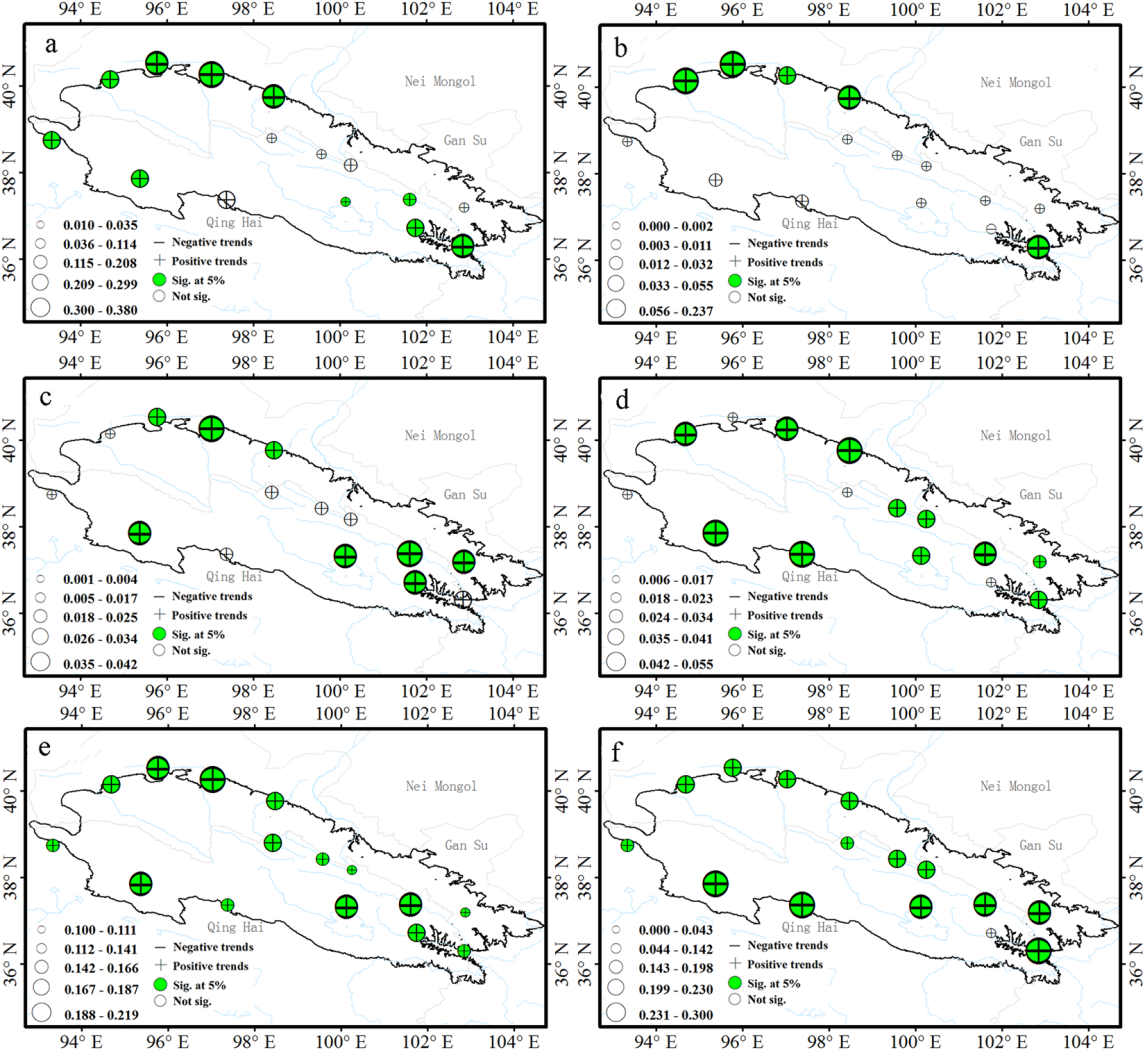
Spatial pattern of indices of warm-temperature extremes. Warm indices: summer days (**a**), tropical nights (**b**), warmest days (**c**), warmest night (**d**), warm days (**e**), warm nights (**f**). For description of indices, see Supplementary Table S1. The size of the circles is the degree of changing. Positive trends are shown as pluses, negative trends as minuses. A green symbol indicates significance at the 5%; a white symbol represents non-significant trends. The maps are generated with Arc Map Ver 10.1 (http://www.esri.com/software/arcgis/arcgis-for-desktop).

Between 40 and 100% of stations showed statistically significant increases for the indices of warm extremes (Fig. 1). For the number of summer days with >25 °C daily maximum temperature (SU 25), 67% of stations showed significant and increasing trends mainly in the northwestern and eastern parts of the Qilian Mountains (Figs 1 and 2). Similarly, increasing trends were observed in the number of tropical nights with the daily minimum temperature of >20 °C (TR 20); 40% of stations (Tmin < 20 °C in six stations) with increasing trends significant at the 5% level were mainly distributed in the northern part of the study area (Figs 1 and 2). For the warmest day of the year (TXx), and the warmest night of the year (TNx), approximately 54 and 74% of stations, respectively, exhibited significant increasing trends (Figs 1 and 2). Further, some indices showed significant increasing trends in all stations; these included warm days, defined as the percent of days when daily maximum >90^th^ percentile (TX90p), and warm nights, defined as percent of days when daily minimum >90^th^ percentile (TN90p) (Fig. 2).

**Figure 3:**
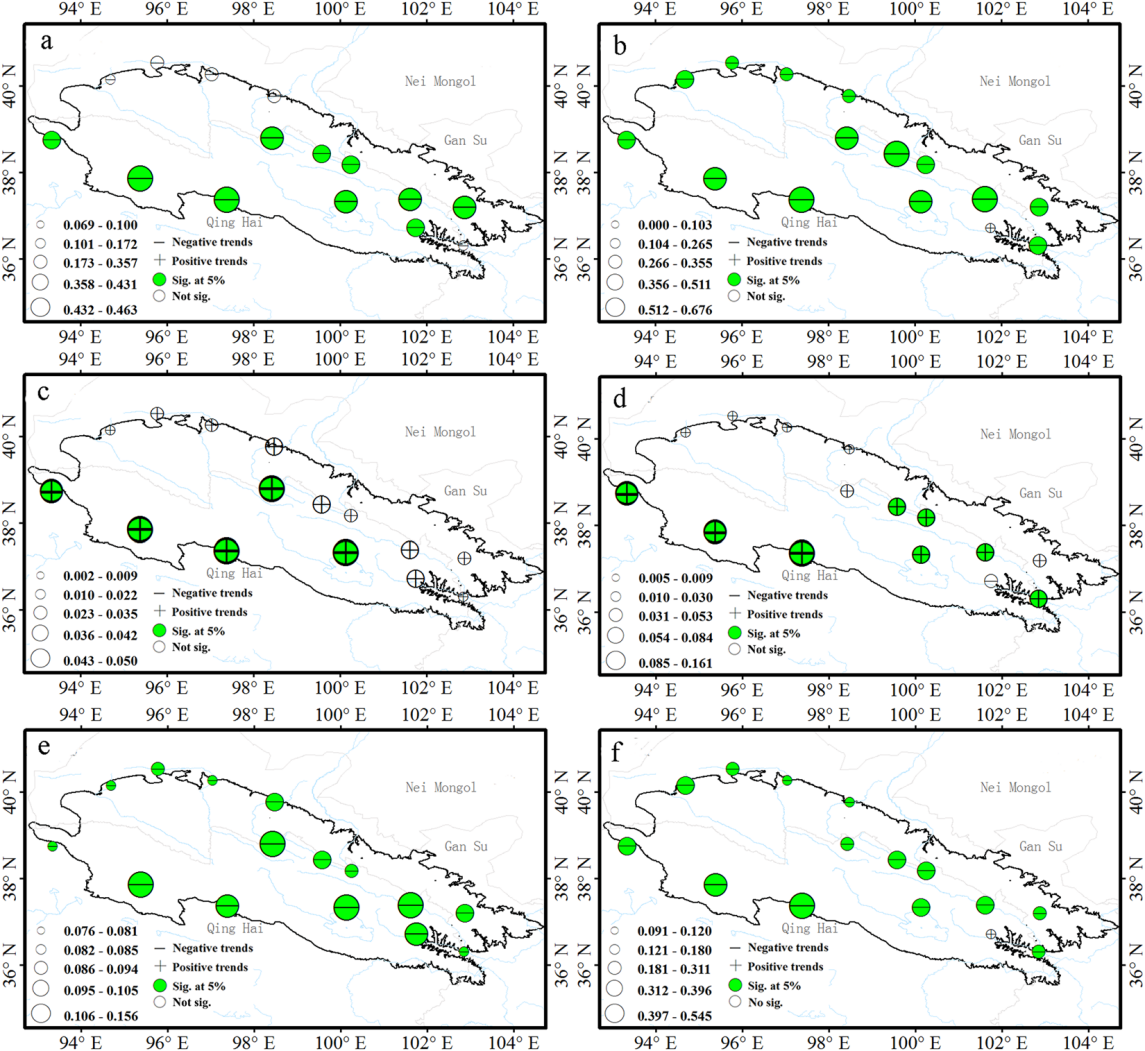
Spatial pattern of indices of cold-temperature extremes. Cold indices: ice days (**a**), frost days (**b**), coldest day (**c**), coldest night (**d**), cool days (**e**), cool nights (**f**). For description of indices, see Supplementary Table S1. The size of the circles is the degree of changing. Positive trends are shown as pluses, negative trends as minuses. A green symbol indicates significance at the 5%; a white symbol represents non-significant trends. The maps are generated with Arc Map Ver 10.1 (http://www.esri.com/software/arcgis/arcgis-for-desktop).

Almost all of the stations in the Qilian Mountains experienced significantly positive trends in the indices of cold temperature extremes during the past 50 years (Figs 1 and 3). All stations showed decreasing trends in the number of ice days (ID0), with 67% being significant; and stations were not significant concentrated mainly in the northwest (Figs 1 and 3). All stations exhibited significant decreasing trends for the number of frost days (FD0), except the Xining station (Fig. 3). Temperatures of the coldest days (TXn) and coldest nights (TXn) showed increasing trends at almost all stations except Xining, which was decreasing, and approximately 34 and 54% of stations, were statistically significant, respectively (Figs 1 and 3). Due to the effect of human activities and urbanization, the Xining station showed different trends than observed elsewhere. The percentage of days when the maximum and minimum temperature was less than the 10th percentile (TX10p and TN10p) exhibited decreasing trends, and that was statistically significant for 100 and 94% of the stations, respectively for TX10p and TN10p (Fig. 3).

### Temporal trends in temperature extremes

**Figure 4:**
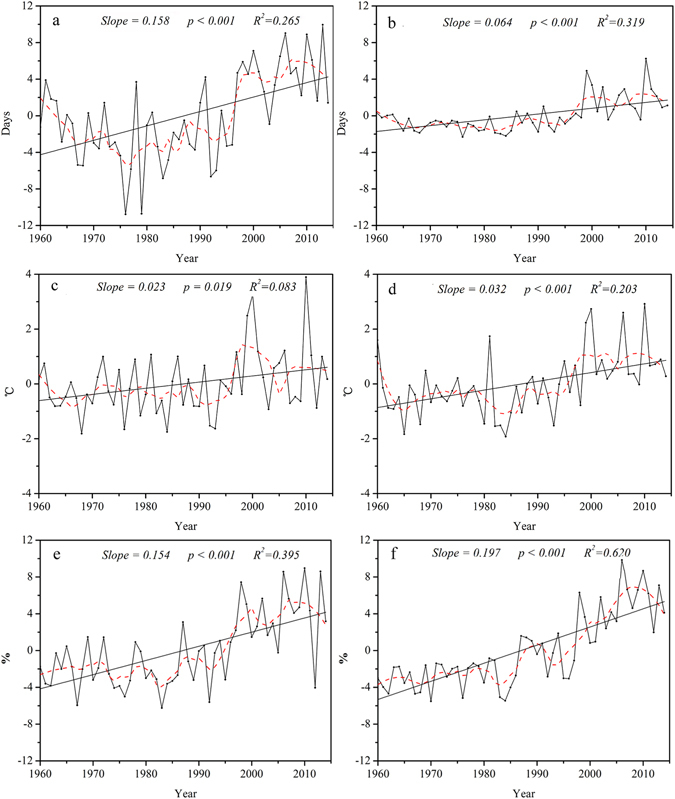
Annual regional averaged series for warm extremes. Warm indices: summer days (**a**), tropical nights (**b**), warmest days (**c**), warmest night (**d**), warm days (**e**), warm nights (**f**). For description of indices, see Supplementary Table S1. Straight line represents the linear regression and the dash line is the 10-year smoothed average.

**Figure 5:**
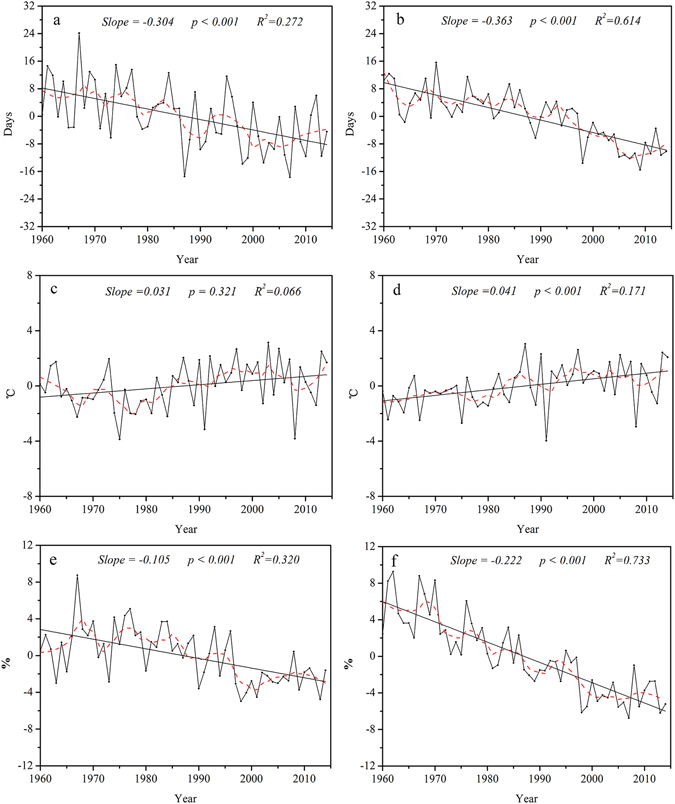
Annual regional averaged series for cold extremes. Cold indices: ice days (**a**), frost days (**b**), coldest day (**c**), coldest night (**d**), cool days (**e**), cool nights (**f**). For description of indices, see Supplementary Table S1. Straight line represents linear regression and the dash line is the 10-year smoothed average.

The regional averaged time series of indices of temperature extremes in the Qilian Mountains are shown in Figs 4 and 5.

The regional averaged number of days per decade increased by 1.58 (*P* < *0.01*) for summer days (SU 25), and 0.64 (*P* < *0.01*) for tropical nights (TR 20) (Fig. 4). The regional averaged trends in the maximum and minimum daily temperature (TXx and TNx) increased with the rate of 0.23 and 0.32 °C/decade, and were statistically significant (Fig. 4). Similarly, the regional averaged increase in the percentage of days exceeding the 90th percentiles (TX90p and TN90p) was 1.54 / decade for warm days and 1.97 /decade for warmest nights (Fig. 4). On the whole, the regional trends in warm temperature indices revealed increases in the daily minimum rather than the daily maximum temperatures in recent decades.

In addition, most of the indicators of warm extremes showed large variation during 2000–2014, including number of tropical night (TR 20), maximum temperature (TXx), and percentage of days with TX > 90^th^ percentile (TX90p) (Fig. 4). This indicated that the frequency of occurrence of maximum and minimum temperature extremes increased under the influence of the changing climate that means the climate was warm and dry in the past, and then changed to warm and wet^26^ due to the average temperature has risen by 0.26 °C/ decade and annual precipitation by 7.6 mm / decade in this region in the past 50 years^27^.

In contrast to warm extremes, indices that were related to cold extremes showed significant decreasing trends (Fig. 5). In particular, the regional averaged number of ice days (ID0) and frost-free days (FD0) significantly decreased from 1960 to 2014 by −3.04 and −3.63 days/decade (Fig. 5). Number of cool days (TX10p) and cool nights (TN10p) decreased by 1.05 and 2.22 / decade, respectively (Fig. 5). The lowest annual daily maximum (TXn) and minimum temperatures (TNn) exhibited slightly increasing trends (Fig. 5). This suggested that the increase in extreme temperature comprises both an increase in the extreme maximum and extreme minimum temperatures. Cold and warm extremes exhibited a consistent trend in that daily minimum was greater than daily maximum temperature (Figs 4 and 5).

Most of the indicators exhibited significant changes with seasons. Warmest day and warmest night (TXx and TNx) exhibited warming in every season, with significant trends in winter. Percentage of warm days and nights (TX90p and TN90P), and the lowest values of maximum and minimum temperatures (TXn and TNn) increased as well. However, summer and winter showed the greatest number of significant trends. For instance, significant warming trends were observed for daily mean maximum temperature (TXM) and daily mean minimum temperature (TNM) in winter, with rates of 0.99 and 1.55 °C/decade, respectively. It was noteworthy that the rate of TNM was greater than that of TXM. These results revealed that overall increases in mean temperatures may be attributed mostly to increases in mean minimum temperatures (TNM), particularly in winter.

### Diurnal temperature range (DTR) and the duration indices (GSL, WSDI, CSDI)

**Figure 6:**
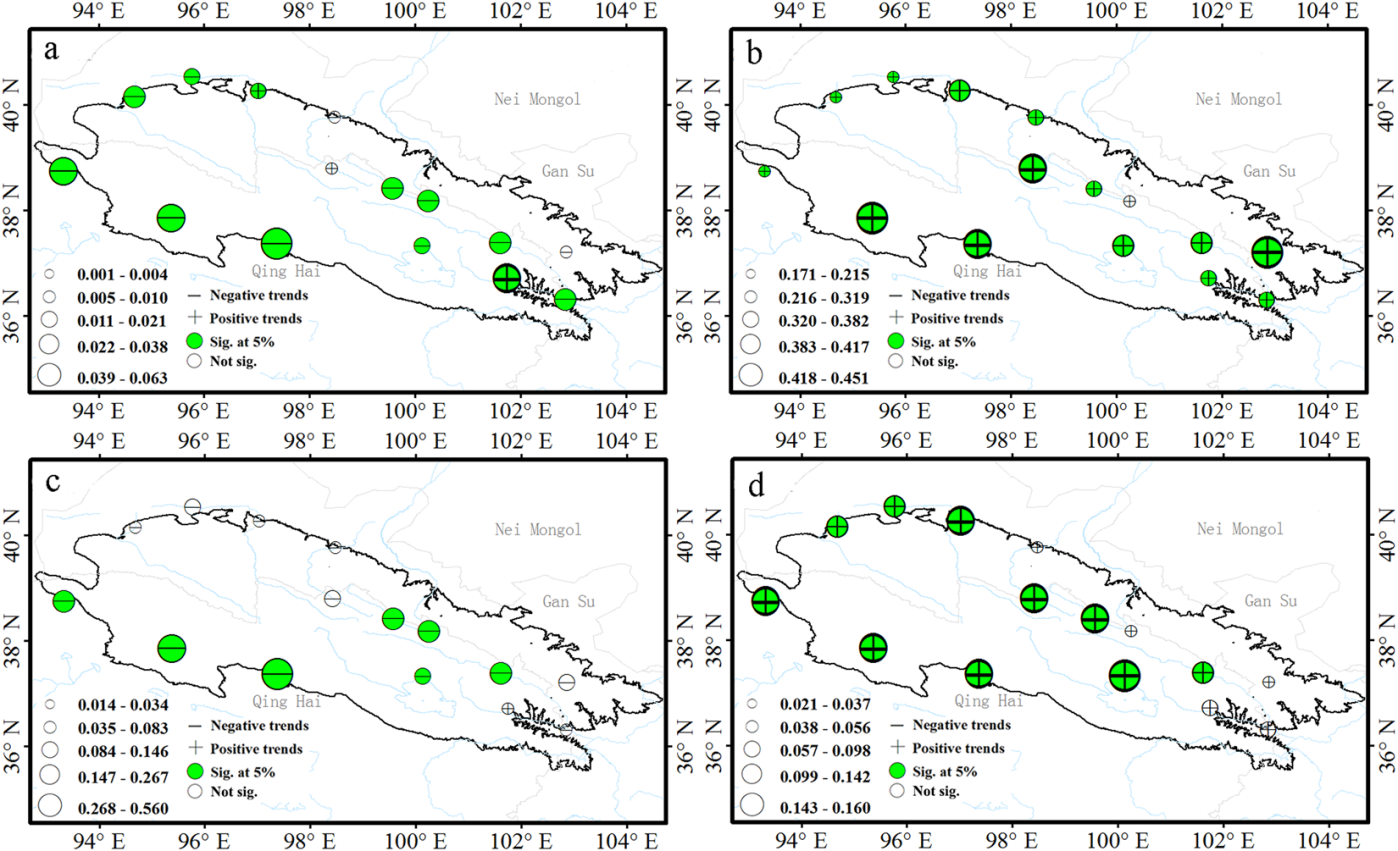
Spatial pattern of diurnal temperature range and duration indices. Diurnal temperature range (**a**), growing season length (**b**), cold spell duration (**c**), warm spell duration (**d**). For description of indices, see Supplementary Table S1. The size of the circles is the degree of changing. Positive trends are shown as pluses, negative trends as minuses. A green symbol indicates significance at the 5%; a white symbol represents non-significant trends. The maps are generated with Arc Map Ver 10.1 (http://www.esri.com/software/arcgis/arcgis-for-desktop).

**Figure 7:**
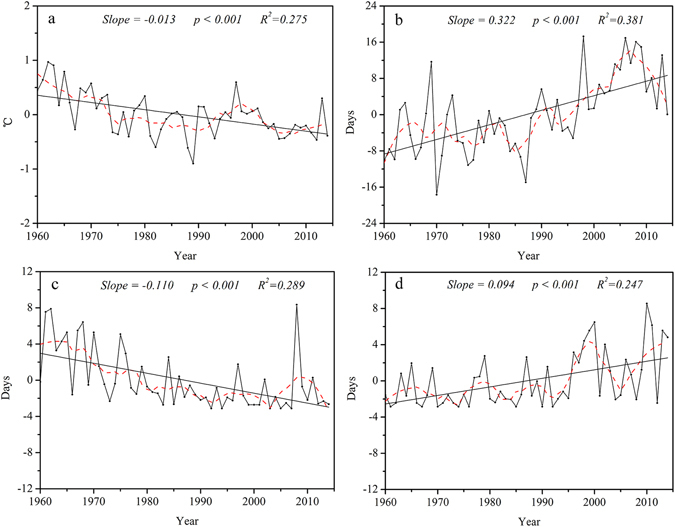
Annual regional averaged series for diurnal temperature range and the duration indices. Diurnal temperature range (**a**), growing season length (**b**), cold spell duration (**c**), warm spell duration (**d**). For description of indices, see Supplementary Table S1. Straight line represents linear regression and the dash line is the 10-year smoothed average.

DTR, GSL, WSDI and CSDI are the duration indices reflected the time interval. Most stations showed significant decreasing trends in diurnal temperature range (DTR); however, 20% of stations exhibited an increase in DTR (Figs 1 and 6). For example, urban environment of Xining and Yumen are surrounded by farmland in the past gradually change to buildings and cement ground in the Urbanization Process. It leads to the increasing rate of daily mean maximum temperature was greater than that of mean minimum temperatures and exhibited an increase trends of diurnal temperature range in Xining and Yumen stations. The average regional diurnal temperature range decreased at a rate of −0.13 °C/decade (P < 0.01) (Fig. 7). In addition, DTR exhibited a significant (P < 0.01) decreasing trend in every season (Table 1). This was further evidence that the increasing rate of TNM was greater than that of TXM.

**Table 1:** Seasonal trends in average monthly indices from 1960–2014.

	TXM	TNM	DTR	TXx	TNx	TX90p	TN90P	TXn	TNn	TX10p	TN10p
Spring	0.06*	0.10**	−0.02**	0.06*	0.07*	0.30*	0.44**	0.02	0.11**	−0.21*	−0.51**
Summer	0.07**	0.12**	−0.05**	0.07*	0.10**	0.55**	0.46**	0.05*	0.14**	−0.20*	−0.78**
Fall	0.10**	0.11**	−0.02**	0.09**	0.10**	0.47**	0.56**	0.11*	0.16**	−0.48**	−0.65**
Winter	0.10**	0.16**	−0.06**	0.11**	0.17**	0.54**	0.62**	0.10*	0.15**	−0.38*	−0.73**

For description of index names, see Supplementary Table S1. TXM: daily mean maximum temperature, TNM: daily mean minimum temperature. For description of index names, see Supplementary Information S2. * and ** denote significance at the 0.05 and 0.01 level, respectively.

All stations showed positive trends for the growing-season length (GSL), which increased from 1.71 to 4.51 days/decade; this trend was significant for 93% of stations (Fig. 6). Trends across the region from 1960 to 2014 exhibited a sharp increase at a rate of 3.22 days/decade (Fig. 7).

The duration of cold spell (CSDI) decreased by 1.10 days/decade (*P* < *0.01*) over the past 50 years (Fig. 7). Rates of −0.14 to −5.40 days/decade were significant for 47% of stations (Figs 1 and 6), located mainly in the southwest and mid-areas. In addition, warm spell-duration indicator (WSDI) increased by 0.94 days/decade (*P* < *0.01*) (Fig. 7), with 67% of stations exhibiting significant increases with rates of 0.20 to 1.60 days/decade (Figs 1 and 6).

### Correlation coefficients of extreme temperature indices

Results revealed that extreme temperature indices correlated well with the daily mean temperature (P < 0.01) (Table 2). The warm-related indices were positively correlated with the daily mean temperature, except for diurnal temperature range (R = −0.24); correlation coefficients ranged from 0.45 to 0.93. Warm days (TX90p) and warm nights (TN90p) exhibited the highest correlation with the daily mean temperature (R = 0.89 and 0.93, respectively).

**Table 2:** Correlation coefficients for extreme temperature indices in the Qilian Mountains.

	TM	SU25	TR20	ID0	TX10p	TX90p	TN10p	TN90p	CSDI	WSDI	FD0	GSL	DTR	TXx	TNx	TXn	TNn
TM	1																
SU25	0.69**	1															
TR20	0.49**	0.79**	1														
ID0	−0.83**	−0.42**	−0.29*	1													
TX10p	−0.84**	−0.64**	−0.39**	0.75**	1												
TX90p	0.89**	0.74**	0.52**	−0.71**	−0.65**	1											
TN10p	−0.93**	−0.57**	−0.44**	0.76**	0.80**	−0.71**	1										
TN90p	0.93**	0.72**	0.58**	−0.69**	−0.65**	0.93**	−0.80**	1									
CSDI	−0.49**	−0.25*	−0.26*	0.47**	0.43**	−0.27*	0.65**	−0.33**	1								
WSDI	0.61**	0.63**	0.61**	−0.47**	−0.44**	0.75**	−0.51**	0.68**	−0.26*	1							
FD0	−0.89**	−0.56**	−0.39**	0.60**	0.68**	−0.77**	0.88**	−0.87**	0.42**	−0.54**	1						
GSL	0.73**	0.52**	0.28*	−0.45**	−0.62**	0.69**	−0.68**	0.74**	−0.24*	0.36*	−0.84**	1					
DTR	−0.24*	0.04	−0.16	0.06	−0.13	−0.07	0.42**	−0.33**	0.35**	−0.05	0.45**	−0.22	1				
TXx	0.45**	0.84**	0.81**	−0.25*	−0.40**	0.48**	−0.39**	0.48**	−0.26*	0.52**	−0.31*	0.20	−0.01	1			
TNx	0.48**	0.69**	0.85**	−0.21	−0.33**	0.50**	−0.45**	0.58**	−0.28*	0.48**	−0.44**	0.34**	−0.25*	0.74**	1		
TXn	0.43**	0.26*	0.15	−0.47**	−0.56**	0.32**	−0.43**	0.26*	−0.46**	0.25*	−0.32**	0.20**	0.19	0.11	0.07	1	
TNn	0.46**	0.22	0.17	−0.49**	−0.41**	0.37**	−0.47**	0.32**	−0.53**	0.28*	−0.34**	0.12**	−0.05	0.12	0.13	0.84**	1

For description of index names, see Supplementary Table S1. TM is daily mean temperature. * and ** represent significance at the 0.05 and 0.01 level, respectively.

Cold extremes exhibited significant negative correlations with the daily mean temperature ranging from −0.49 to −0.93. Cold nights (TN10p) exhibited the highest correlation (R = −0.93). Furthermore, indices correlated well among themselves. For example, the highest correlation coefficients existed between the number of summer days (SU 25) and the maximum temperature (warmest day; TXx) (R = 0.84), and between the number of tropical nights (TR 20) and warm nights (TN90p) (R = 0.85).

### Spatial trends in precipitation extremes

**Figure 8:**
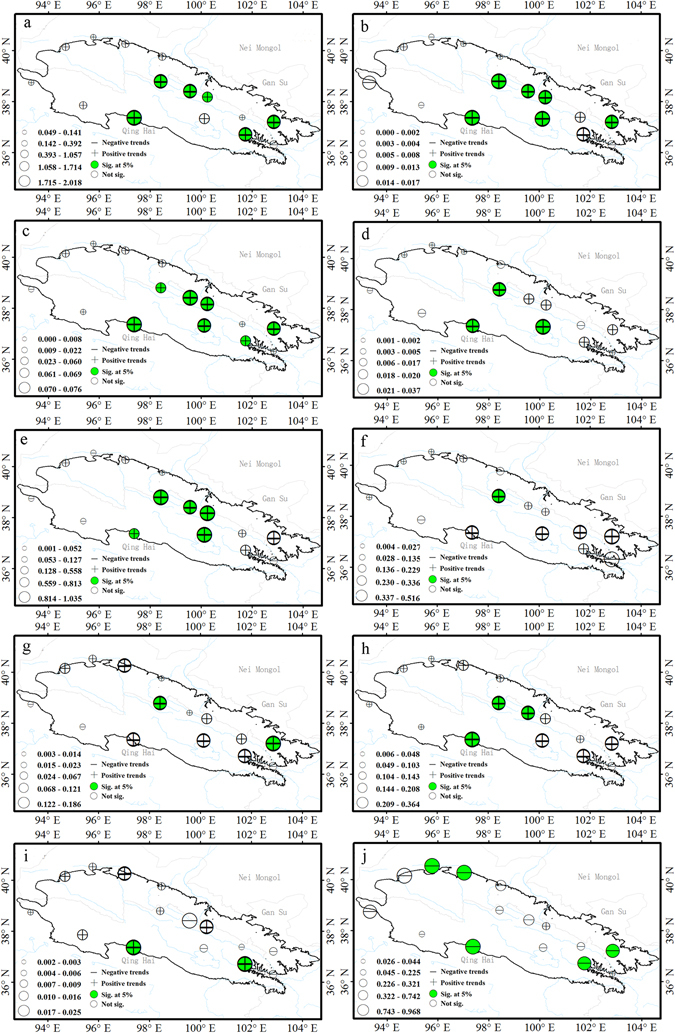
Spatial patterns of indices of precipitation extremes. Precipitation indices: wet day precipitation (**a**), simple daily intensity (**b**), heavy precipitation days (**c**), very heavy precipitation days (**d**), very wet day precipitation (**e**), extremely wet days precipitation (**f**), maximum 1-day precipitation (**g**), maximum 5-day precipitation (**h**), consecutive wet days (**i**), consecutive dry days (**j**). For description of indices, see Supplementary Table S1. The size of the circles is the degree of changing. Positive trends are shown as pluses, negative trends as minuses. A green symbol indicates significance at the 5%; a white symbol represents non-significant trends. The maps are generated with Arc Map Ver 10.1 (http://www.esri.com/software/arcgis/arcgis-for-desktop).

Spatial distribution of trends in indices of precipitation extremes are shown in Fig. 8.

Almost all precipitation indices, except the number of consecutive dry days (CDD), exhibited increasing trends across the region; however, only a fraction was statistically significant.

About 93% of the stations exhibited increases in annual total precipitation (PRCPTOT) over the past 50 years, with 40% being significant at the 5% level (Fig. 1). Areas experiencing significant increases were central and eastern; those areas exhibited the largest change in amplitudes as well (Fig. 8). The simple daily intensity (SDII) increased at 67% of stations, indicating that precipitation events had become more intense across the region (Figs 1 and 8).

The number of days with heavy (R10mm) and very heavy precipitation (R20mm) exhibited significant increasing trends at approximately 47 and 20% of stations, respectively, which were concentrated in the central part of the high altitude areas. Increases in very wet (R95p) and extremely wet precipitation (R99p) were noted at 73 and 80% of the stations, respectively; only 33% were significant at the 5% level for R95p and 7% for R99p, with distribution similar to that of annual total precipitation (PRCPTOT; Figs 1 and 8). The maximum 1-day (Rx1 day) and maximum 5-day precipitation (Rx5 day) showed increasing trends at approximately 80 and 93% of stations, respectively (Figs 1 and 8). Overall, there were few significant trends.

The 20% of stations with significant decreasing trends in consecutive dry days (CCD) were located mainly at the edge of the Qilian Mountains. In contrast, the consecutive wet days (CWD) exhibited no clear spatial trend and few significant trends. The distribution characteristics suggest that, over time, the dry spells became shorter, while the wet spells became longer for most stations.

### Temporal trends in precipitation extremes

**Figure 9:**
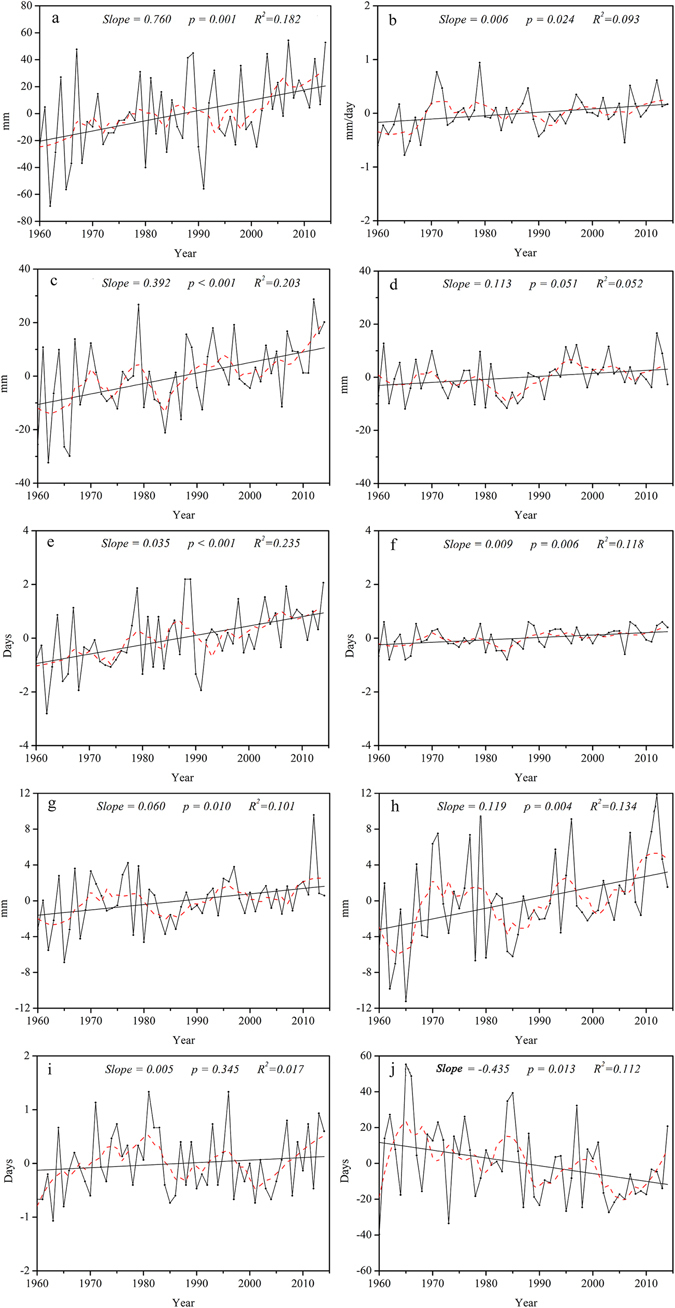
Annual regional averaged series for indices of precipitation extremes. Precipitation indices: wet day precipitation (**a**), simple daily intensity (**b**), heavy precipitation days (**c**), very heavy precipitation days (**d**), very wet day precipitation (**e**), extremely wet days precipitation (**f**), maximum 1-day precipitation (**g**), maximum 5-day precipitation (**h**), consecutive wet days (**i**), consecutive dry days (**j**). For description of indices, see Supplementary Table S1. Straight line represents linear regression and the dash line is the 10-year smoothed average.

The annual regional averaged total wet-day precipitation (PRCPTOT) and simple daily intensity (SDII) exhibited increasing trends from 1960 to 2014, at rates of 7.60 mm/ decade and 0.24 mm/day/decade, respectively (Fig. 9). In addition, maximum 1-day (Rx1 day) and 5-day precipitation (Rx5 day), and precipitation on very wet (R95p) and extremely wet days (R99p) increased, as well. However, the trend for precipitation on extremely wet days (R99p) was not significant.

The regional increasing trend in heavy (R10mm) and very heavy precipitation days (R20mm) paralleled those of other indicators, but the magnitudes and ranges differed (Fig. 9). R10mm and R20mm increased at rates of 0.35 days/decade and 0.09 days/decade, respectively.

The duration indices, consecutive wet (CWD) and dry days (CDD), differed in trends across the region. There was a non-significant increase in CWD, and a significant decrease in CDD at rate of −4.35 days/decade (Fig. 9).

In general, consistency among indices suggested that average simple daily intensity and precipitation on wet days increased along with the amount of precipitation on extreme precipitation days and periods, but consecutive dry days decreased (Fig. 9). This indicated a decrease in the longest dry spell, corresponding in most instances to dry season length rather than a dry spell in the rainy season.

### Correlation coefficients of precipitation indices

The extreme precipitation indices, except for consecutive dry days (R = −0.29), were highly correlated with total annual precipitation, with correlation coefficients >0.5, and statistical significance level of 0.01 (Table 3).

**Table 3:** Correlation coefficients of extreme precipitation indices in the Qilian Mountains.

	PRCPTOT	SDII	RX1 day	RX5 day	R95p	R99p	CDD	CWD	R10mm	R20mm
PRCPTOT	1									
SDII	0.71**	1								
RX1 day	0.71**	0.67**	1							
RX5 day	0.68**	0.59**	0.81**	1						
R95p	0.81**	0.83**	0.85**	0.73**	1					
R99p	0.56**	0.59**	0.83**	0.66**	0.74**	1				
CDD	−0.29*	−0.12	−0.20	−0.25*	−0.17	−0.12	1			
CWD	0.50**	0.29*	0.36**	0.64**	0.34**	0.14	−0.24*	1		
R10mm	0.92**	0.81**	0.65**	0.61**	0.83**	0.54**	−0.29*	0.41**	1	
R20mm	0.73**	0.77**	0.75**	0.62**	0.90**	0.76**	−0.13	0.25*	0.75**	1

For description of index names, see Supplementary Table S1. * and ** represent significance at the 0.05 and 0.01 level, respectively.

Extreme precipitation indices were also correlated. For instance, heavy precipitation days (R10mm) and simple daily intensity (SDII) was highly positively correlated (R = 0.81).

## Discussion

Climate extremes is exacerbating and triggering due to global warming, including decreasing the frequency of cold days and cold nights and increasing the frequency of warm days and warm nights^1^. An arid mountain region is a sensitive area to global warming due to its unique physiographic characteristics and geographic location. The detailed analysis of the characteristics of climate extremes, represented by the variability of those climate extreme indices, substantiates the general understanding of the association of regional climate change to global warming, and thus should contribute to get a deeper understanding of the complex interactions between the regional climate variety and the global climate change^28^.

We used 55-years of daily records from 15 stations in the Qilian Mountains to calculate 16 extreme temperature and 10 extreme precipitation indices. Specifically, we summarized positive and negative trends, and the regional averaged trends, and analyzed the spatial changes in these indices. This information can be used to identify indicators that are most representative of extreme climate trends across the Qilian Mountains^5^.

Our results indicated increasing trends in temperature and precipitation extremes between 1960 and 2014. The highest increases in daily mean minimum temperature, daily mean maximum temperature, warm days, warm nights, coldest day, coldest night, warmest day and night were observed in winter months. This is consistent with reports from the Loess Plateau^3^ and the arid areas of northwestern China^29^. These results reveal that overall increases in mean temperatures may be attributed mostly to increases in the mean minimum temperatures, particularly in winter. Because the average minimum increased more than the maximum temperature, the diurnal temperature range (DTR) also decreased. Many studies indicated that DTR decreased under climate change, but the decrease differed among different areas. Thus, DTR decreased by 0.06 °C/decade in the Loess Plateau^3^, and 0.26 °C/decade in Xinjiang^30^ both in China; the greatest decrease was reported in summers for the southeastern United States^7^.

Growing season length (GSL) is the most important factor for tree-ring growth in the Qilian Mountains^19^. We found that all stations showed positive trends in GSL, some with substantial increases of 3.22 days/decade. Previous studies in the region revealed a 3.7 days/decade increase in GSL in *Picea crassifolia* forests during 2001–2011^22^. Increases in growing season length may benefit ecological processes, for example, increasing in growing season length may affect forest productivity, and make the wood production and carbon sinks increased in the long term^31, 32^, but have negative effects on the hydrologic cycle in the Qilian Mountains; for instance, transpiration in *P. crassifolia* forests increased by 3–4% as a result of the expansion of the growing season^25^.

All of the precipitation indices, except consecutive dry days, exhibited increasing trends for the whole region. The distribution characteristics suggested that dry spells became shorter, and wet spells became longer for most stations. The trends in precipitation indices generally agree with previous research in other regions; for example, increases in precipitation extremes were noted for the Loess Plateau^3^ and Songhua River Basin^12^ of China, and the southeastern United States^7^. The decrease in consecutive dry days indicated that, rather than the length of dry spells in the rainy season, the length of the dry season decreased. Increasing amount of precipitation contributed to the changes in precipitation extreme events.

These results are consistent with reports for the southeastern United States^7^, Europe^33^, arid areas of northwestern China^29^, the Loess Plateau of China^3^, the Yangtze River region during 1962–2011^34^, and globally^6^. However, the change magnitudes of extreme indices are not quite similar. The reasons for the above results may be an arid mountain region is a sensitive area to global warming due to its unique physiographic characteristics and geographic location. Climate extremes is exacerbating and triggering in the arid mountain region due to global warming.

We conclude that the frequency or magnitudes of extreme climatic events exhibit increasing trends across the Qilian Mountain region and support previous predictions that the climate in northwestern China is changing from cold-dry to warm-wet^26^. The impacts of climatic change in an arid mountain region are likely to have significant repercussions not only on ecological and hydrologic processes of the mountains, but also on populated downstream regions that depend on mountain water resources for domestic, agricultural, energy, and industrial purposes.

The climate trends imply higher rates of evaporation, and a greater proportion of solid to liquid precipitation (snow to rainfall)^35^; these physical mechanisms (degradation of permafrost, available soil water increased)^36^, associated with potential changes in precipitation amount and temperature seasonality, will affect soil moisture, plant growth, groundwater reserves, and the frequency of flood or drought episodes in an arid mountain region. Thus how to gain an in-depth understanding of this complexity is an important item on our future research agenda.

## Data and Methods

### Study area

The Qilian Mountains are located at in the northeastern margin of the Tibetan Plateau, adjoining the Badain Jaran Desert and the Tengger Desert to the north, the Qaidam Basin to the south, the Taklimakan Desert and the Tarim Basin to the west and the Loess Plateau to the east^37^. The area is composed of several parallel mountains and valleys, and stretches for about 850 km east to west, and 200–300 km north to south. The Qilian Mountains are in the transition zone between the influence of the East Asian Monsoon and the Westerlies. Climate is semi-arid to arid, continental mountain in the temperate zone, with cold and dry winters under the influence of the Mongolian anticyclone^38^. The annual mean temperature ranges from 6 °C at lower elevations to −5 °C at higher elevations. The annual precipitation ranges from 150 mm in the foothills to 800 mm in the high mountains and about 85% of the annual precipitation falls in the growing season from May to September^39^. Several inland rivers originate in the Qilian Mountains from precipitation and snowmelt water.

### Data sources and quality controls

This study utilized data from 15 meteorological stations that recorded daily precipitation, and maximum and minimum temperatures in the Qilian Mountains [Details of the selection of meteorological stations are in Supplementary Figure S1]. The data were provided by the National Climate Center of the China Meteorological Administration (http://www.nmic.gov.cn).

Data quality was checked with RClimDex package (software and documentation available for download from http://etccdi.pacificclimate.org/software.shtml), which can automatically check for errors (e.g., negative precipitation, maximum temperature less than or equal to minimum temperature). Homogeneity assessments of the recorded daily temperature and precipitation data sets were conducted by software packages (software and documentation available for download from http://etccdi.pacificclimate.org/software.html): RHtests for temperature and RHtests-dlyprcp for precipitation series. The purpose of this assessment is to detect artificial shifts due to inevitable changes in the instruments, relocations, environment, and procedures during data collection^40^. The application consists of two steps: first, all missing values are replaced into an internal format that the software recognizes (i.e. NA, not available), but time series with more than 20% missing values within the analysis period 1960–2014 were excluded, and if precipitation values were below 0 mm, or minimum temperature exceeds maximum temperature, or observation were more than four standard deviations from the mean, they were replaced into NA. And then using the RHtest and RHtests-dlyprcp to perform the two phase regression to detect multiple step change points that could exist in a time series, which is a method to assess data homogeneity^40^. RHtests was applied based on the penalized maximal t test and the penalized maximal F test, which are embedded in a recursive testation algorithm, with the lag-1 autocorrelation of the time series being empirically accounted for. The RHtests-dlyPrcp was similar to the RHtests, except that it was specifically designed for the homogenization of daily precipitation data time series. It was based on the transPMFred algorithm, which integrates a data-adaptive Box–Cox transformation procedure into the PMFred algorithm^40^. After quality control and homogeneity assessments, RClimDex was used to calculate indices of extreme climate from the daily data.

### Extreme indices

This study used 26 extreme-climate indices, including 16 extreme temperature and 10 extreme precipitation indices [Details of the selection of extreme-climate indices are in Supplementary Table S1]. These indices were selected from the core list of indices developed by the Expert Team on Climate Change Detection and Indices (ETCCDI)^40^. Temperature indices included 9 warm-weather related, and 7 cold-weather related. Precipitation indices included 9 wet-weather related and 1 dry-weather related. These indices have been extensively used to assess changes in extreme temperature and precipitation events because they can reflect changes in intensity, frequency, and duration of high/low temperature and precipitation events^3, 4, 6, 7, 27, 41, 42^.

### Methods

The simple linear regression method was employed to analyze trends for the selected indices of weather extremes at the 15 meteorological stations for regional averaged series. The nonparametric Mann–Kendall trend test, commonly used to assess the significance of monotonic trends in hydro-meteorological time series^43–45^, was used to evaluate the statistical significance of the linear trends.

The Mann–Kendall test statistic (S) is given by

1 $$S=\sum _{k=1}^{n-1}\sum _{j=k+1}^{n}{\rm{sgn}}({x}_{j}-{x}_{k})$$

and

2 $$\begin{array}{c}if\,\theta  > 0,\,\mathrm{sgn}(\theta )=1\\ if\,\theta =0,\,sgn(\theta )=0\\ if\,\theta  < 0,\,\mathrm{sgn}(\theta )=-\,1\end{array}$$

where *n* is the data set record length and *x*_*j*_ and *x*_*k*_ are the sequential data values.

The Mann–Kendall test has two parameters that are important for trend detection. They are: the significance level, which indicates the trend’s strength, and the slope magnitude estimate, which indicates the direction as well as the rate of change. Under the null hypothesis, there is no trend in the data, and the distribution *S* is expected to have a mean of zero and a variance of

3 $${\rm{var}}(S)=\frac{n(n-1)(2n+5)}{18}$$

The normal Z-test statistic is calculated as

4 $$z=\{\begin{array}{c}\frac{S-1}{\sqrt{{\rm{var}}(S)}}\,if\,S > 0\\ 0\,\,\,\,if\,S=0\\ \frac{S+1}{\sqrt{{\rm{var}}(S)}}\,if\,S < 0\end{array}$$

The null hypothesis is rejected at a two-sided significance level of *p* if |Z| > Z_(*1*−*p/2*)_, where Z(_*1*−*p/2*_) is the value of the standard normal distribution with a probability of exceeding *p/2*. A positive value of Z indicates an upward trend, while a negative value represents a downward trend in the data.

If a linear trend is present, the magnitude of the trend, *β*, or the slope (change per unit time) is estimated using a non-parametric method proposed by Sen^46^ and extended by Hirsch *et al*.^47^:

5 $$\beta =Median[\frac{{X}_{j}-{X}_{k}}{j-k}]\,for\,all\,k < j$$

where *1* < *k* < *j* < *n*, or the slope estimator *β* is the median of all possible combinations of pairs for the whole data set.

The regional averaged series for each index was calculated as follows:

6 $${x}_{r,t}=\sum _{i=1}^{{n}_{t}}({x}_{i,t}-{\overline{x}}_{i})/{n}_{t}$$

where *x*_*r,t*_ is the regional averaged index for year *t*; *x*_*i,t*_ is the index for station *i* in year *t*; *x*_*i*_ is the index mean at station *i* over the period 1960–2014; *n*_*t*_ is the number of stations with data in year *t* ^4^.

Changes in the occurrence of temperature extremes during certain times of the year can have important implications for some regions^7^. Therefore, we investigated seasonal trends in climatic extremes to reveal more detail about intra-annual dynamics (Table 1). Seasons were defined as spring (March to May), summer (June to August), fall (September to November), and winter (December to February).

The correlation analysis was used to examine the relationships between extreme temperature indices and the daily mean temperature, and extreme precipitation indices and total annual precipitation.

## Electronic supplementary material

Supplementary Figure S1 and Supplementary Table S1
