# Supplementary material for: Recent changes in daily climate extremes in an arid mountain region, a case study in northwestern China’s Qilian Mountains
Source: Sci Rep. 2017 May 22;7:2245. doi: 10.1038/s41598-017-02345-4 (PMC5440392; doi:10.1038/s41598-017-02345-4)
Supplement: Supplementary file 1 — Supplementary Figure S1 and Supplementary Table S1 [file 41598_2017_2345_MOESM1_ESM.pdf]

Supplementary Information on

Recent changes in daily climate extremes in an arid mountain region, a case study in northwestern China's Qilian Mountains

PengFei Lin[1,2], ZhiBin He [1,*], Jun Du [1], LongFei Chen [1],Xi Zhu[1,2], Jing Li[1,2]

[1] *Linze Inland River Basin Research Station, Chinese Ecosystem Research Network, Key Laboratory of Eco-hydrology of Inland River Basin, Northwest Institute of Eco-Environment and Resources, Chinese Academy of Sciences, Lanzhou 730000, China.*[2] *University of Chinese Academy of Sciences, Beijing 100049, China*

*Corresponding author address: Zhibin He, Northwest Institute of Eco-Environment and Resources, Chinese Academy of Sciences, Lanzhou 730000, China. E-mail: hzbmail@lzb.ac.cn

Supplementary Figure S1

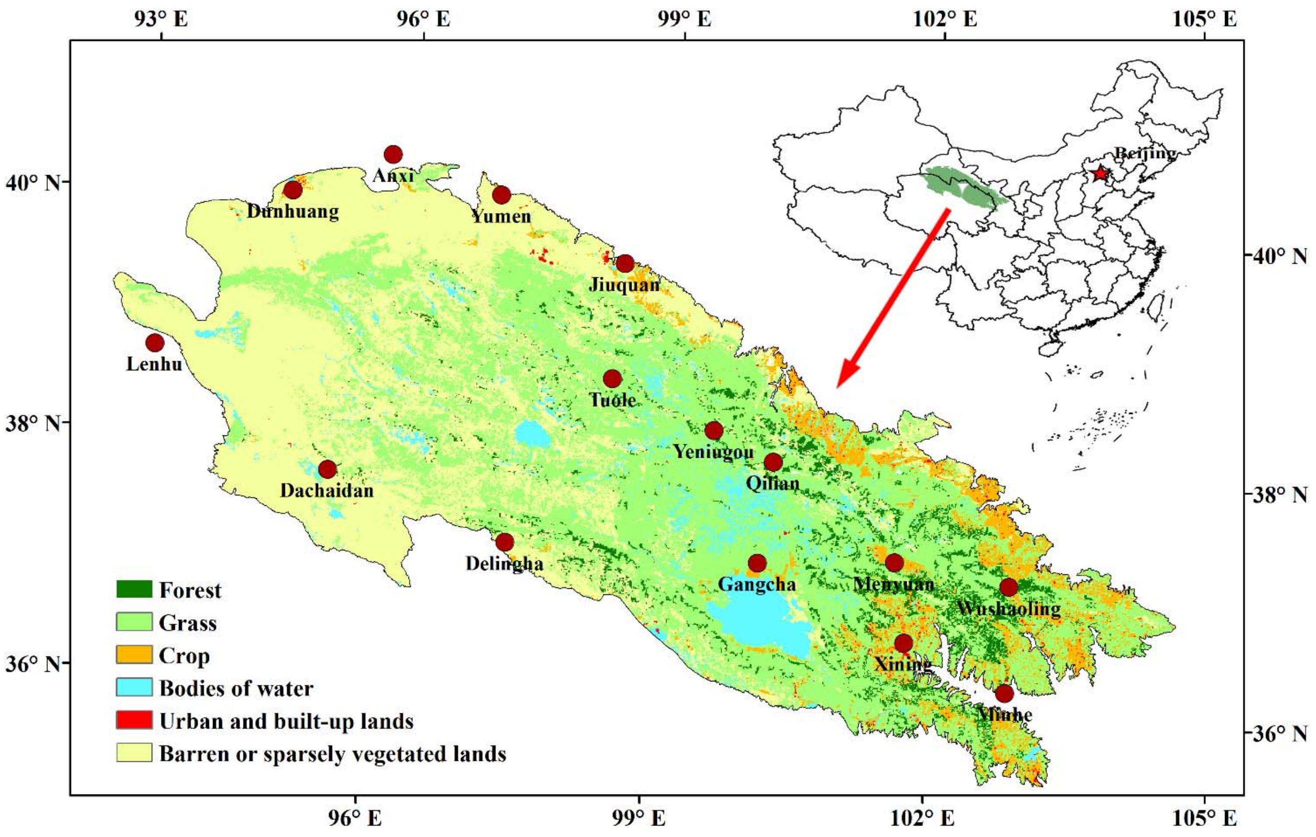

Fig. 1. Locations of meteorological stations (red circles) in the Qilian Mountains. The maps are generated with Arc Map Ver 10.1 (http://www.esri.com/software/arcgis/arcgis-for-desktop).

Supplementary Table S1

Table 1. Indices of temperature and precipitation extremes.

Temperature Indices			
Index	Descriptive Name	Definition	Units
**Warm-Related Indices**			
SU25	Summer days	Annual count when TX > 25℃	days
TR20	Tropical nights	Annual count when TN < 20℃	days
TXx	Warmest day	Maximum value of TX per month	℃

TNx	Warmest night	Maximum value of TN per month	℃
DTR	Diurnal temperature range	Mean monthly difference between TX and TN	℃
TX90p	Warm days	Percentage of days when TX > 90th percentile	%
TN90p	Warm nights	Percentage of days when TN > 90th percentile	%
WSDI	Warm spell duration	Annual count of days over at least 6 consecutive days when TX>90th percentile	days
GSL	Growing season length	Annual count between first span of at least 6 days with TM > 5℃ after winter and first span after summer of 6 days with TM < 5℃	days
**Cold-Related Indices**			
ID0	Ice days	Annual count when TX < 0℃	days
FD0	Frost days	Annual count when TN < 0℃	days
TXn	Coldest day	Minimum monthly value of TX	℃
TNn	Coldest night	Minimum monthly value of TN	℃
TX10p	Cool days	Percentage of days when TX < 10th percentile	%
TN10p	Cool nights	Percentage of days when TN < 10th percentile	%
CSDI	Cold spell duration	Annual count of days with at least 6 consecutive days when TN < 10th percentile	days

Precipitation Indices			
Index	Descriptive Name	Definition	Units
**Wet Indices**			
R10mm	Heavy precipitation days	Annual count of days when RR ≥ 10mm	days

R20mm	Very heavy precipitation days	Annual count of days when RR ≥ 20mm	days
Rx1day	Maximum 1-day precipitation	Maximum annual 1-day precipitation	mm
Rx5day	Maximum 5-day precipitation	Maximum annual consecutive 5-day precipitation	mm
R95p	Very wet day precipitation	Annual total precipitation when RR>95th percentile	mm
R99p	Extremely wet days precipitation	Annual total precipitation when RR>99th percentile	mm
CWD	Consecutive wet days	Maximum number of consecutive wet days	days
PRCPTOT	Wet day precipitation	Annual total precipitation from wet days	mm
SDII	Simple daily intensity index	Average precipitation on wet days	mm/d
**Dry Index**			
CDD	Consecutive dry days	Maximum number of consecutive dry days	days

TX is the daily maximum temperature; TN is daily minimum temperature; TM is daily mean temperature; RR is the daily precipitation. A wet day is defined when RR >= 1 mm; a dry day is when RR < 1 mm. All indices were calculated on an annual basis from January to December.
